# Gingerol-Rich Extract Derived from *Zingiber officinale* Roscoe Alleviates Motion Sickness via Inhibiting the Ileal IL-33/ST2/PLC-γ1/TRPA1 Pathway

**DOI:** 10.3390/ijms27146124

**Published:** 2026-07-08

**Authors:** Longhui Yan, Ziming Xia, Yiming Luo, Junyu Bu, Kai Liang, Chang Liu, Xin Sun, Zhiyan Zhang, Min Li, Shuchen Liu, Ying Tian

**Affiliations:** Academy of Military Medical Sciences, Beijing 100850, China; yanlonghui111@163.com (L.Y.); zmxia22@163.com (Z.X.); 13137200855@163.com (Y.L.); bujunyu112@163.com (J.B.); a2724963261@163.com (K.L.); liuchang6926@163.com (C.L.); sunx0102@163.com (X.S.); zzyan0911@163.com (Z.Z.); limin82057@163.com (M.L.); liusc118@163.com (S.L.)

**Keywords:** gingerol-rich extract, motion sickness, neuromodulation, 5-HT, Fos, IL-33/ST2/PLC-γ1/TRPA1 signaling pathway

## Abstract

Gingerol-rich extract derived from *Zingiber officinale* Roscoe (ZOGE) is clinically effective against motion sickness (MS), yet its mechanism remains unknown. Here, we demonstrate that ZOGE confers protection by modulating a specific gut–brain immune–neuroendocrine pathway. Using a rotation-induced rat model combined with behavioral tests, vestibular nuclei (VN) metabolomics, and molecular analyses, we found that ZOGE not only alleviated MS symptoms and normalized VN metabolic disturbances, particularly in phenylalanine, tyrosine, and tryptophan biosynthesis, but also potently suppressed the peripheral IL-33/ST2/PLC-γ1/TRPA1 signaling axis in the ileum, leading to reduced synthesis and release of 5-HT, a key MS mediator. Our study provides the evidence that ZOGE acts through coordinated central metabolic modulation and peripheral inhibition of a defined pro-emetic pathway, establishing a novel gut–brain immune–neuroendocrine mechanism for its therapeutic efficacy against MS.

## 1. Introduction

Motion sickness (MS) is an autonomic disorder provoked by sensory conflict among visual, vestibular (inner ear), and proprioceptive (body position) systems, frequently experienced during air and space travel, sea voyages, and automotive transportation. Its clinical presentation encompasses both autonomic manifestations (nausea, vomiting, hypersalivation, and pallor) and non-autonomic symptoms (metabolic alterations, fatigue, and lethargy) [1,2]. Conventional pharmacotherapies for MS (e.g., antihistamines and anticholinergics), though effective for prophylaxis, are often limited by adverse effects such as sedation, dry mouth, blurred vision, and cognitive dysfunction. These drawbacks restrict their use among individuals in high-alertness professions, including pilots, sailors, and drivers [2]. As a result, there is growing interest in plant-derived therapeutic alternatives for managing MS.

A gingerol-rich extract derived from *Zingiber officinale* Roscoe (ZOGE), a class of active ingredients of ginger-derived pungent compounds in a traditional Chinese medicine against MS, is proven to be clinically effective in alleviating nausea and vomiting, through multi-target mechanisms. Its beneficial effects are mediated via physiological modulation of a neurotransmitter-associated signaling pathway [3,4], gastrointestinal function [5,6,7], gut microbiota [8], and anti-inflammation and antioxidative properties [9]. Notably, ZOGE exhibits a favorable safety profile without sedative effects, making it a promising candidate for populations requiring sustained alertness and vulnerable groups. Nevertheless, its precise mechanisms of action against MS remain incompletely understood.

The prevailing hypothesis suggests that MS arises from sensory conflicts between actual visual/vestibular inputs and neural predictions of motion, highlighting the pivotal role of the vestibular system. Motion signals processed by vestibular organs are transmitted to the vestibular nucleus (VN) within the medullopontine region, which plays a central role in the pathogenesis of MS. Rodent models have shown that provocative motion stimuli activate VN neurons, leading to autonomic responses indicative of MS, including piloerection, salivation and conditioned taste aversion [1]. Investigations of c-fos protein (Fos) expression further support VN involvement, demonstrating neuronal activation in the caudal VN following rotational stress—an effect mitigated by vestibular lesion [10]. Importantly, nausea and vomiting, evolutionarily conserved responses for toxin expulsion, involve the release of gut-derived serotonin (5-hydroxytryptamine, 5-HT) from enterochromaffin cells (ECs) via the IL-33/ST2/PLC-γ1/TRPA1 signaling pathway, which orchestrates intestinal homeostasis and emetic defense [11,12]. IL-33, a new member of the IL-1 cytokine family, is known to exhibit increased expression in the central nervous system in response to inflammatory stimuli [13]. Jafarzadeh et al. have reported that ginger extract positively regulates both IL-33 and IL-27 in the central nervous system of experimental autoimmune encephalomyelitis mice [14]. TRPA1 channels are non-selective cation channels that can be activated by ginger-derived pungent compounds, contributing to improving antioxidant effects and the digestive function [15,16]. However, it remains unclear whether MS engages the gut–brain immune–neuroendocrine axis, and whether ZOGE alleviates MS by modulating the IL-33/ST2/PLC-γ1/TRPA1 signaling pathway.

This study aimed to investigate the neuromodulated mechanisms underlying the anti-MS effects of ZOGE. Using a well-established animal model of MS, we evaluated the efficacy of ZOGE, performed metabolomic profiling of VN to identify associated metabolic pathways, and experimentally validated candidate signaling mechanisms through biochemical and molecular assays.

## 2. Results

### 2.1. ZOGE Mitigates MS Symptoms and Restores Metabolic Homeostasis in a Rotation-Induced Rat Model

**Figure 1:**
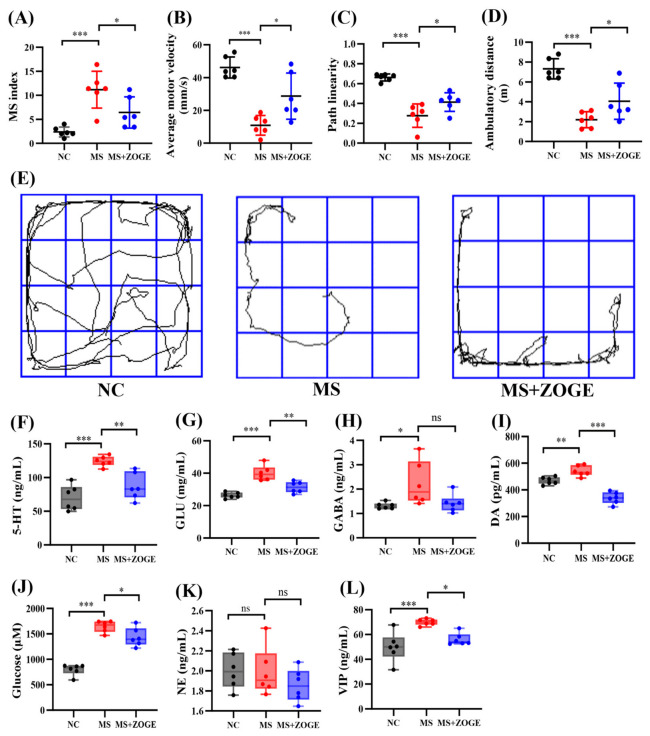
Effect of ZOGE on rotation-induced MS symptoms and serum biomarkers in rats. (**A**) MS index, (**B**) average motor velocity, (**C**) path linearity, (**D**) ambulatory distance. (**E**) The representative locomotor tracks. Serum biomarker contents of 5-HT (**F**), GLU (**G**), GABA (**H**), DA (**I**), glucose (**J**), NE (**K**), and VIP (**L**). Data are presented as mean ± SD. *** *p* < 0.001, ** *p* < 0.01, * *p* < 0.05, and ns indicates non-significant compared with the MS group.

Rats exposed to rotational stimulation exhibited classic autonomic symptoms of MS, including piloerection, generalized tremor, and increased fecal and urinary excretion. In the absence of an emetic reflex, these animals display accelerated intestinal transit and fecal expulsion to alleviate gastric load and visceral discomfort. The MS index, a well-validated composite score, was used to quantify the severity of MS. Provocative rotation significantly increased the MS index (Figure 1A; *p* < 0.001 vs. NC), whereas pretreatment with ZOGE markedly attenuated this increase (*p* < 0.05), demonstrating its protective effect against MS.

An open field test (OFT) further revealed significant sensorimotor impairment in MS rats, as indicated by decreased average velocity (*p* < 0.001), reduced path linearity (*p* < 0.001), and shortened ambulatory distance (*p* < 0.001). These behavioral changes reflect disrupted vestibular-motor integration and autonomic dysfunction. ZOGE treatment effectively reversed these impairments, restoring motor velocity, path linearity, and total distance to levels comparable to those of control animals (Figure 1B–E; all *p* < 0.05 vs. MS). 

Systemic metabolic analysis using quantitative ELISA assays showed that rotational stress induced significant elevations in serum of 5-HT (*p* < 0.001), glutamic acid (GLU, *p* < 0.001), γ-aminobutyric acid (GABA, *p* < 0.05), dopamine (DA, *p* < 0.01), glucose (*p* < 0.001), and vasoactive intestinal peptide (VIP, *p* < 0.001) without affecting the concentration of norepinephrine (NE) (Figure 1F–L). Administration of ZOGE selectively normalized the metabolic disturbances of 5-HT, GLU, DA, glucose, and VIP. Together, these results collectively suggest that ZOGE may alleviate MS-related symptoms and metabolic dysregulation by modulating the brain–gut neuroendocrine axis.

### 2.2. Metabolomic Profiling of VN Reveals ZOGE-Dependent Modulation in Rotation-Induced MS Rats

**Figure 2:**
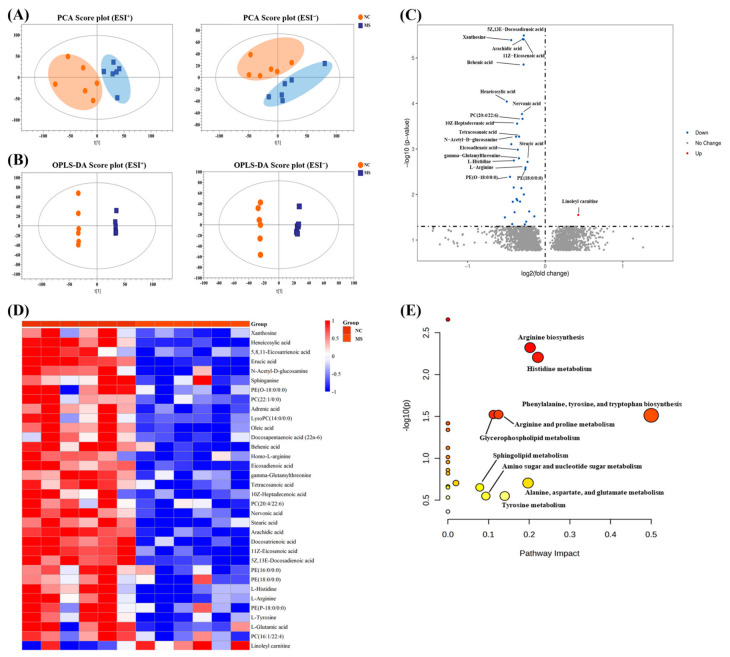
Identification and pathway analysis of differential metabolites between NC and MS groups. (**A**) PCA score plot in positive and negative ion mode: (ESI^+^, R^2^X = 0.459, Q^2^ = 0.128) and (ESI^−^, R^2^X = 0.615, Q^2^ = 0.203). (**B**) OPLS-DA score plot in positive and negative ion mode: (ESI^+^, R^2^Y = 1, Q^2^ = 0.854) and (ESI^−^, R^2^Y = 0.998, Q^2^ = 0.705). (**C**) Volcano plot showing differentially expressed metabolites with fold change. Red and blue plot dots separately mean upregulation and downregulation in the NC group compared with the MS group. (**D**) Heatmap of differential metabolites in VN tissue. The color is proportional to the intensity of change in metabolites. (**E**) Summary of pathways with MataboAnalyst.

Metabolic changes in the VN of rats were evaluated after one hour of rotational stimulation. Principal component analysis (PCA) incorporating quality control (QC) samples was first performed to assess system stability and experiment reliability. The pooled QC samples clustered tightly in both positive and negative ion modes (Figure S1), demonstrating the high instrument stability and data quality of the UPLC-Q-TOF-MS platform. Total ion current (TIC) chromatograms from untargeted metabolomic profiling of VN tissues across the three experimental groups are shown in Figure S2. To investigate rotation-induced metabolic alterations, PCA was used to compare the NC and MS groups, revealing clear separation between them and indicating substantial metabolic disruption in the VN following rotational stress (Figure 2A). Subsequently, orthogonal partial least squares-discriminant analysis (OPLS-DA) was applied to improve feature selection and identify differentially abundant metabolites. The OPLS-DA model demonstrated strong predictive capability, with cumulative R^2^Y and Q^2^ values exceeding 0.7 (Figure 2B). Differential metabolites between the NC and MS groups were selected based on a variable importance in projection (VIP) score > 1 from the OPLS-DA model and a *p*-value < 0.05 from Student’s *t*-test. In total, 34 metabolites were significantly altered, involving 10 in positive ion mode and 24 in negative ion mode. These included six amino acid-related metabolites, 16 free fatty acids, eight glycerophospholipids, and four compounds from other classes (Figure 2C and Table 1). A heatmap visualization illustrates distinct expression patterns of these metabolites in the VN following rotational stimulation (Figure 2D). Pathway analysis using MataboAnalyst (v6.0) identified the three most significantly affected metabolic pathways, phenylalanine, tyrosine, and tryptophan biosynthesis, histidine metabolism, and arginine metabolism (Figure 2E), indicating their potential roles in the vestibular response to rotational challenge.

**Figure 3:**
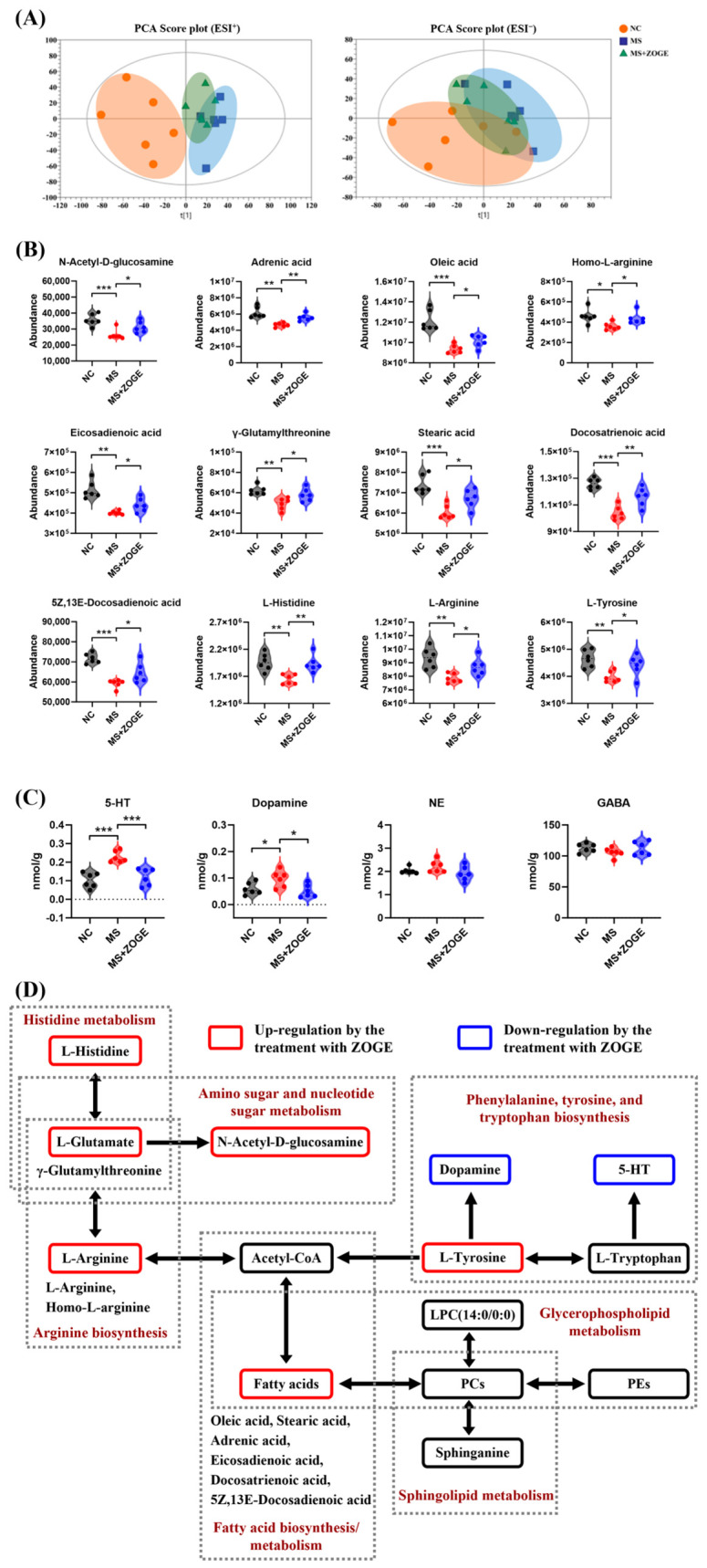
Effect of ZOGE on differential metabolites and pathways in rotation-induced MS rats. (**A**) PCA score plot in positive and negative ion mode: (ESI^+^, R^2^X = 0.593, Q^2^ = 0.231) and (ESI^−^, R^2^X = 0.63, Q^2^ = 0.26). (**B**) Differentially expressed metabolites in VN regulated by ZOGE treatment. (**C**) Target neurotransmitter quantification in VN among the NC, MS, and MS + ZOGE groups. (**D**) Important network of metabolic pathways regulated by ZOGE treatment. Data are presented as mean ± SD. *** *p* < 0.001, ** *p* < 0.01, * *p* < 0.05.

To clarify the regulatory influence of ZOGE on the VN in rats under rotational stress, multivariate analysis was used to assess metabolic differences among the three experimental groups. As shown in Figure 3A, a distinct separation was observed between the MS and NC groups, while the MS + ZOGE group clustered closer to the NC group, indicating that ZOGE treatment partially reverses rotation-induced metabolic alterations in the VN. Furthermore, ZOGE administration promoted the restoration of 12 differentially expressed metabolites toward normal levels (Figure 3B). 

Moreover, neurotransmitter profiling within the VN showed that NE and GABA levels were non-significant among the three experimental groups (Figure 3C). However, 5-HT and DA levels of rat VN in the MS group were significantly increased after the rotational stimulation. ZOGE treatment effectively reduced 5-HT and DA concentrations in the VN to baseline levels, which were consistent with the modulatory effects on serum 5-HT and DA. Based on these differentially expressed metabolites and neurotransmitters, a metabolic regulatory network of ZOGE against MS was constructed (Figure 3D), highlighting roles in amino acid and lipid metabolisms. These results implied that 5-HT, a key mediator in phenylalanine, tyrosine, and tryptophan biosynthesis, may serve as a potential biomarker for the exploration of the anti-MS mechanism. 

### 2.3. ZOGE Modulates the MEK/CREB/Fos Pathway in the VN of Rotation-Induced MS Rats

**Figure 4:**
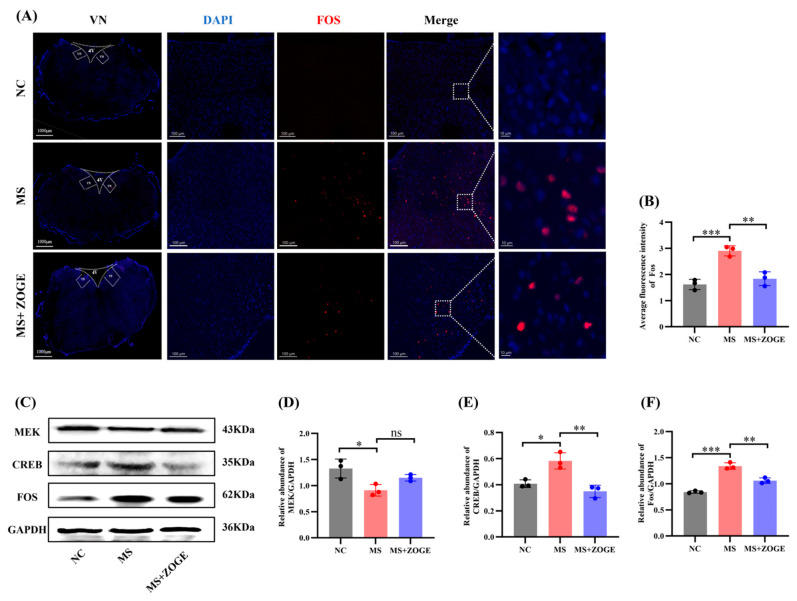
Effect of ZOGE on the expression of Fos in the VN of rotation-induced MS rats. (**A**) Representative immunofluorescence image of Fos (red) and DAPI-stained nuclei (blue). (**B**) Fos expression in rat VN was analyzed using ImageJ software. (**C**) Representative images of protein expressions in the VN tissues. GAPDH served as the internal control. (**D**–**F**) Gray value statistics of corresponding proteins. Data are presented as mean ± SD. *** *p* < 0.001, ** *p* < 0.01, * *p* < 0.05, and ns indicates non-significant compared with the MS group.

To further elucidate the neuromodulating mechanisms of ZOGE, we assessed the expression of Fos and associated signaling proteins within the VN. Immunofluorescence (IFC) staining and Western blotting analysis demonstrated a significant upregulation of Fos expression in the VN of the MS group compared to the NC group (*p* < 0.001, Figure 4A–C,F). ZOGE treatment markedly reduced Fos expressions in rotation-induced rats (*p* < 0.01). Furthermore, rotational stress led to decreased MEK and increased CREB protein levels in the VN (*p* < 0.05, Figure 4C–E). Administration of ZOGE displayed an increasing tendency in the MEK protein level (*p* > 0.05) and significantly downregulated the CREB protein level (*p* < 0.01), showing regulatory effects on the MEK/CREB/Fos pathway in the VN of rotation-induced MS rats. 

### 2.4. ZOGE Reduces Ileal 5-HT Levels in Rotation-Induced MS Rats by Inhibiting the IL-33/ST2/PLC-γ1/TRPA1 Signaling Pathway

**Figure 5:**
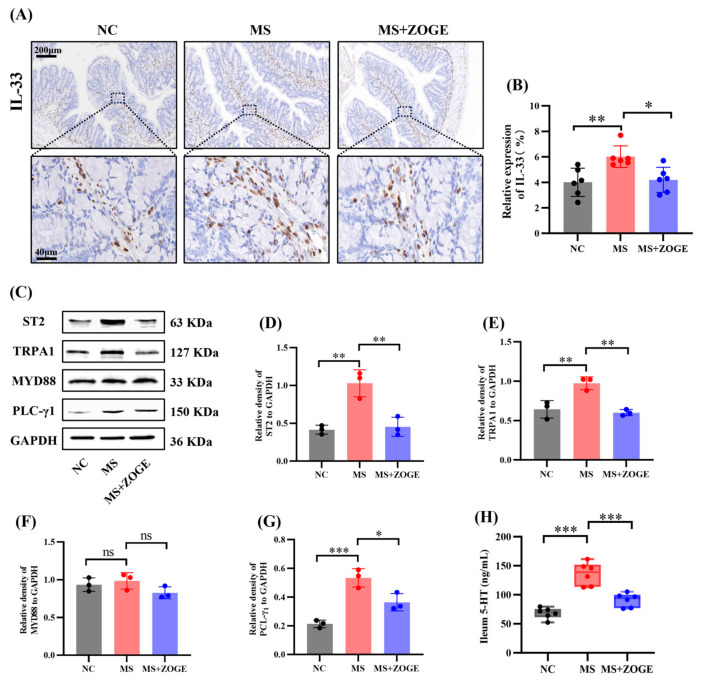
Effect of ZOGE on the ileal IL-33/ST2 signaling pathway in rotation-induced MS rats. (**A**) Representative images of IL-33. (**B**) Quantification of IL-33 expression levels. (**C**) Representative ST2, TRPA1, MYD88, PLC-γ1, and GAPDH images by Western blotting in ileal. (**D**–**G**) Gray value statistics of corresponding proteins. (**H**) The levels of 5-HT in ileal. Data are presented as mean ± SD. *** *p* < 0.001, ** *p* < 0.01, * *p* < 0.05, and ns indicates non-significant compared with the MS group.

Previous studies demonstrate that activation of the IL-33/ST2/PLC-γ1/TRPA1 signaling pathway in ECs stimulates 5-HT secretion, contributing to nausea and vomiting. We therefore hypothesized that this pathway may also mediate MS-induced nausea and vomiting. As shown in Figure 5, rotational stimulation significantly increased the expression of ileal signaling proteins, including IL-33, ST2, PLC-γ1, and TRPA1, while MYD88 expressions remained unaltered, indicating the involvement of a non-canonical IL-33/ST2/PLC-γ1/TRPA1-mediated pathway (Figure 5A–G). ZOGE treatment effectively suppressed the expression of these proteins. Correspondingly, ileal 5-HT levels were significantly higher in the MS group than in the NC group, whereas the MS + ZOGE group showed a pronounced reduction in 5-HT (Figure 5H). These findings imply that ZOGE alleviates MS-related nausea and vomiting by suppressing 5-HT release and inhibiting the IL-33/ST2/PLC-γ1/TRPA1 signaling pathway.

### 2.5. ZOGE Inhibits Ileal 5-HT-Associated Proteins in Rotation-Induced MS Rats

**Figure 6:**
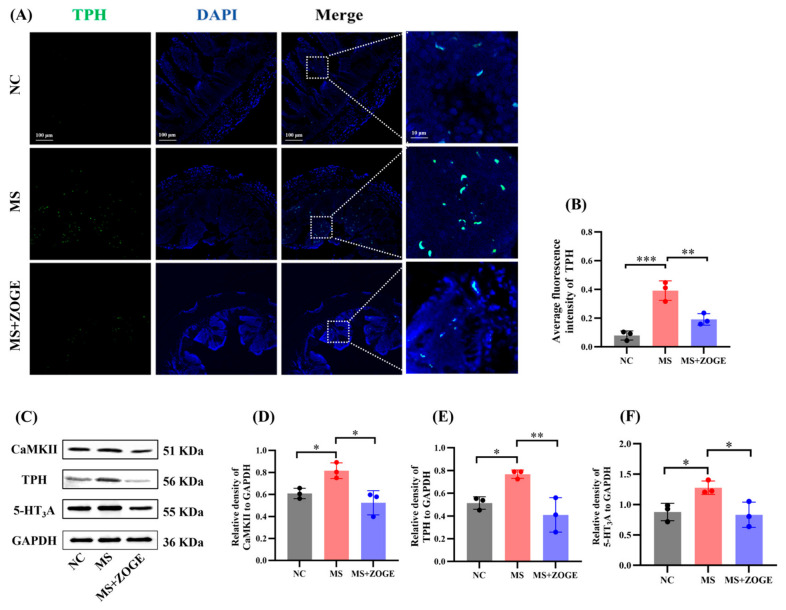
Effect of ZOGE on the expression of proteins in ileal 5-HT biosynthesis and signaling in rotation-induced MS rats. (**A**) Representative immunofluorescence image of TPH (green) and DAPI-stained nuclei (blue). (**B**) Quantitative analysis of TPH protein level using ImageJ software. (**C**) Representative CaMKII, TPH, 5-HT3A, and GAPDH images by Western blotting in ileal. (**D**–**F**) Gray value statistics of corresponding proteins. Data are presented as mean ± SD. *** *p* < 0.001, ** *p* < 0.01, * *p* < 0.05.

Tryptophan hydroxylase (TPH), the rate-limiting enzyme in peripheral 5-HT biosynthesis, plays a critical role in modulating gastrointestinal function, and its dysregulation is mechanistically associated with enteric nervous system disorders. IFC analysis of ileal TPH revealed a significant increase in TPH levels in the MS group (*p* < 0.001), which was markedly attenuated by ZOGE treatment (*p* < 0.01, Figure 6A,B). Additionally, rotational stress significantly upregulated the ileal protein levels of CaMKII (*p* < 0.05), TPH (*p* < 0.05), and the 5-HT_3_A receptor (*p* < 0.05). ZOGE treatment effectively downregulated the expression of CaMKII (*p* < 0.05), TPH (*p* < 0.01), and 5-HT_3_A (*p* < 0.05) in the ileum (Figure 6C–F). Collectively, these results demonstrate that ZOGE mitigates gastrointestinal symptoms of MS (e.g., nausea and vomiting) through multiple mechanisms involving 5-HT metabolism, including inhibition of TPH-mediated 5-HT biosynthesis, downregulation of CaMKII-dependent signaling transduction and attenuation of 5-HT_3_A receptor activation.

## 3. Discussion

### 3.1. Neuromodulating Characteristics and Biomarkers of ZOGE Against Rotation-Induced Rats

MS is an unpleasant autonomic physiological response experienced by healthy individuals exposed to passive or illusory motion [1]. Accumulating evidence indicates that Fos not only serves as a classical marker of neuronal activation but also as a critical biomarker in the pathogenesis of MS [17]. In the present study, rotational stimulation upregulated Fos expression in the VN of rats, while oral administration of ZOGE significantly normalized Fos expression and modulated its related signaling proteins (MEK and CREB), concomitant with the alleviation of MS symptoms, including improvements in the MS index, behavioral performance, and serum metabolite profiles.

Untargeted metabolomic analysis of the VN revealed that ZOGE-regulated metabolites were primarily associated with amino acids and lipids metabolism, both of which play well-established roles in neural regulation [3,18]. Notably, pathway analysis highlighted the tryptophan, phenylalanine, and tyrosine metabolism pathway, which governs the synthesis of key neurotransmitters such as 5-HT and DA [19]. Targeted neurotransmitter analysis further confirmed that ZOGE specifically modulated the levels of 5-HT and DA in the VN, underscoring its central neuroregulatory role in mitigating MS through the regulation of these neurotransmitter systems [20,21].

Additionally, metabolomic profiling also revealed an increase in L-histidine levels. Although no differential change in its downstream product histamine was directly detected, these findings preliminarily suggested that ZOGE might promote L-histidine synthesis or inhibit its degradation. This observation was consistent with previous investigations, which demonstrated that ginger extract could reduce plasma histamine levels in mice with MS [3,22], thereby supporting the potential antihistaminergic activity of ZOGE.

### 3.2. Neuroregulatory Mechanism of ZOGE Against MS by Acting on the Gut–Brain Immune–Neuroendocrine Axis

Ginger, long recognized as a traditional remedy for nausea and vomiting, demonstrates prominent efficacy in alleviating MS-related symptoms [23]. Serum metabolite profiling indicated that rotational stress induced alterations in peripheral secretions of 5-HT, DA, GLU, glucose, and VIP, all of which were significantly reversed by ZOGE treatment. Although the neuroactive substances in peripheral blood could not directly reflect the levels of central neurotransmitters, they could serve as indirect indicators of nervous system activity and fully demonstrated that ZOGE had a regulatory effect on the neurological changes induced by MS.

Peripheral 5-HT is predominantly produced by ECs in the gastrointestinal tract and plays a vital role in integrating enteroendocrine, immune, and nervous system functions, which is crucial for maintaining gastrointestinal homeostasis [5,11,24]. Given the regulatory effects of ZOGE on both central and peripheral 5-HT synthesis, this study further revealed that rotational stress activated a non-canonical IL-33/ST2/PLC-γ1/TRPA1 signaling pathway in the rat ileum, promoting 5-HT secretion. ZOGE suppressed ileal 5-HT levels and might alleviate MS-induced nausea and vomiting through multi-target inhibition of 5-HT synthesis, involving the rate-limiting enzyme of TPH, upstream signaling activation of IL-33/ST2/PLC-γ1/TRPA1, downstream 5-HT_3_A receptor activation, and CaMKII-mediated signal transduction. 

Notably, although gingerol is classically recognized as a TRPA1 agonist [16], our observed suppression of IL-33/ST2/PLC-γ1/TRPA1 does not contradict this established view. Under physiological conditions, TRPA1 operates at basal activity, and gingerol increases channel opening probability, producing an agonistic effect consistent with the literature. In the MS model, however, rotational stress-induced inflammation and gut dysmotility lead to aberrant upregulation of the IL-33/ST2 axis, resulting in chronic TRPA1 hyperactivation. Under this elevated pathological baseline, additional stimulation by ZOGE may trigger receptor desensitization and negative feedback, culminating in net inhibition rather than excitation. Furthermore, ZOGE is an extract containing multiple bioactive constituents other than gingerol, some of which may act as TRPA1 antagonists or negative modulators. Collectively, our findings reveal a biphasic regulatory property of ginger-derived compounds and highlight the critical importance of validating target engagement in disease-relevant models.

**Figure 7:**
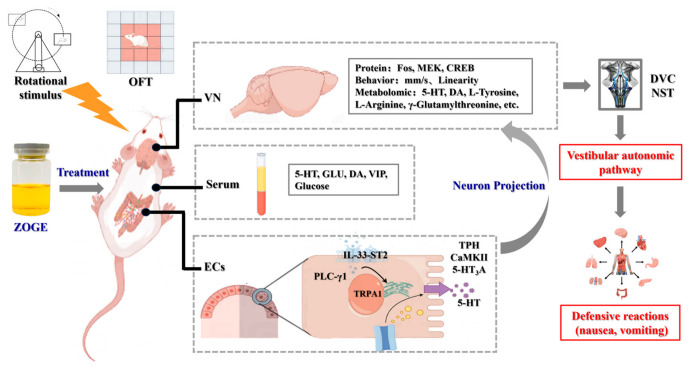
Neuromodulation mechanism of ZOGE against MS.

Taken together, these results suggests that ZOGE engages the gut–brain axis via immune–neuroendocrine networks and influences central autonomic pathways (CREB/MEK/Fos) in the VN through vagal afferent signaling, thereby elucidating part of the neuroregulatory mechanism by which ZOGE ameliorated MS symptoms (Figure 7).

### 3.3. Several Limitations

Although the rotational stimulus-induced rat model is widely used in MS research, we acknowledge its inherent limitation, namely the absence of a vomiting reflex in rats. Consequently, MS assessment in this study relied on surrogate endpoints, including the MS index and OFT behaviors. While these parameters may not fully recapitulate human nausea and vomiting responses, the rat model remains a first-line screening platform for anti-MS agents due to its high throughput, low cost and well-established behavioral readouts. Importantly, the mechanistic cascade revealed in our study provides a foundation for future validation in vomiting-competent species, such as ferrets or pigeons. Furthermore, while ZOGE significantly inhibits the non-canonical pathway regulating 5-HT secretion in ECs, the direct interaction between ZOGE and key target proteins remains unclear. Further studies using techniques such as surface plasmon resonance, cellular thermal shift, or specific inhibitors/agonists are warranted to validate the drug–target interactions and to deepen the mechanistic understanding of ZOGE’s anti-MS effect. Finally, although the IL-33/ST2 signaling pathway-related proteins examined in this study are evolutionarily conserved and highly homologous between rat and mouse models, further attention should be given to evaluating the changes in mouse models of MS to enhance translational relevance.

## 4. Materials and Methods

### 4.1. Materials and Reagents

ZOGE was obtained from Sanjin Group Hunan Sanjin Pharmaceutical Co., Ltd. (Changde, China). ELISA kits for quantitative analysis of 5-HT, NE, VIP, DA, GABA, and GLU were purchased from Nanjing Jiancheng Bioengineering Institute (Nanjing, China).

### 4.2. Animals and Experimental Protocol

Male Sprague-Dawley rats (180–200 g) were sourced from Beijing Vital River Laboratory Animal Technology Co., Ltd. (Beijing, China). Following one week of acclimatization, the rats were randomly assigned to three experimental groups (*n* = 6 per group): a normal control group (NC), a rotational stimulus-induced MS group (MS), and a ZOGE intervention + MS group (MS + ZOGE, 90 mg/kg body weight). ZOGE was dissolved in 0.5% carboxymethylcellulose sodium (CMC-Na) solution. The MS + ZOGE group of rats were orally administrated the ZOGE solution once at 1 h before rotational stimulus. The NC group and the MS group of rats were orally administrated the 0.5% CMC-Na solution at the same time.

### 4.3. Rotational Stimulus

The rotation apparatus (Beijing Zhongshi Dichuang Technology Development Co., Ltd., Beijing, China) used in the present experiment was modified based on the one for cats by Crampton. It consisted of two plexiglass boxes (22.5 × 26 × 20 cm) suspended on a metal frame that revolved about an axis parallel to the floor. Before the rotational stimulus, rats were placed into the plexiglass boxes. Then, rats in the boxes were moved in a clockwise–pause–counterclockwise rotation manner with the acceleration at 16°·s^−2^ to reach 120°·s^−1^ and deceleration at 48°·s^−2^ to reach 0°·s^−1^, which lasted approximately 20 s. During the rotation, the long axis of the rat body was kept perpendicular to the horizontal rotation rod. Rats in the MS group and the MS + ZOGE group received 1 h of rotational stimulus. Simultaneously, rats in the NC group were kept in the chambers close to the rotation apparatus without rotation.

### 4.4. OFT and MS Index Monitor

The OFT (Shanghai Jiliang Software Technology Co., Ltd., Shanghai, China) was conducted in a noncovered, black methacrylate box (100 × 100 × 40 cm) that allowed for video recording during animal testing. After stimulation, rats were individually exposed for 5 min in the OFT. The primary behavioral parameters measured included total ambulatory distance (m), average velocity (mm/s), resting time (s), and movement linearity. During rotation, rats exhibited characteristic symptoms of autonomic nervous system dysfunction, including piloerection, tremors, and increased defecation and urination. Due to absence of vomiting reflex in rats, rats compensate by accelerating intestinal transit and fecal excretion to reduce gastric contents and alleviate discomfort. The MS severity was quantified using a standardized scoring system as follows: feces pellets: each pellet counts for 1 point—if there is no pellet, 0 points; urination: 1.2 points if present, 0 points if not; hair standing up: severe is 1.2 points, mild is 0.6 points, and absence is 0 points; and tremor: 1.2 points if present, and 0 points if not. The MS index is the sum of the scores.

### 4.5. ELISA Assay

5-HT, GLU, GABA, DA, glucose, NE, and VIP of serum were measured using ELISA kits following the manufacturer’s instructions and assessed by recording the absorbance at 450 nm.

### 4.6. UPLC-MS Metabolomics Analysis

The UPLC-MS analysis of the VN samples was performed on a Thermo Dionex UltiMate 3000-Q Exactive system (Thermo Fisher Scientific Inc., Waltham, MA, USA) equipped with an ACQUITY HSS T3 column (2.1 × 100 mm, 1.8 μm; Waters, Milford, MA, USA) at a 50 °C column temperature. The mobile phases consisted of solvent A (0.1% formic acid in water) and solvent B (methanol), and the gradient program was as follows: 0–1 min, mobile phase B at 2.0%; 1–5.5 min, mobile phase B increased from 2.0% to 100%; 5.5–14 min, mobile phase B maintained at 100%; 14–14.1 min, mobile phase B decreased from 100% to 2%; and 14.1–16.0 min, mobile phase B maintained at 2%. The mass spectrometric data were collected with an electrospray ionization (ESI) source operating in positive and negative modes. The optimal conditions were established as follows: capillary temperature at 320 °C, 30 psi of sheath gas pressure, 10 psi of Aux gas pressure, 3.7 kV of source voltage in positive ion mode, and 3.5 kV of source voltage in negative ion mode. The collision gas was nitrogen and its pressure was 1.5 mTorr.

### 4.7. Target Neurotransmitter Quantification

For target metabolomics analysis of 5-HT, GABA, NE, and DA in VN of the rats, approximately 10 mg of sample was mixed with 150 μL methanol. After vortexing and deproteinization at −20 °C for 30 min, the tubes were centrifuged at 15,000× *g* at 4 °C for 10 min. Ten microliters of the supernatant were transferred to a new 1.5 mL centrifuge tube and diluted with 40 μL of 50% methanol solution for further analysis. At the same time, standards (5-HT, GABA, NE, DA) in acetonitrile were processed with the same method for samples as described above. The processed samples were analyzed by the targeted metabolomics technology platform (ACQUITY UPLC-Xevo TQMS, Waters Corp., Milford, MA, USA) for the quantitative analysis of 5-HT, GABA, NE, and DA. All chromatographic separations were performed with an ACQUITY UPLC HSS T3 1.8 μM analytical column (2.1 × 100 mm) (Waters, Milford, MA, USA). The mobile phase consisted of 0.1% (*v*/*v*) formic acid H_2_O solution and 0.1% (*v*/*v*) formic acid acetonitrile solution. Separation of ions mode included the following: 0–1 min (1–15% mobile phase B), 1–2 min (15–19% mobile phase B), 2–3 min (19% mobile phase B), 3–4.9 min (70% mobile phase B), 4.9–5 min (70–100% B), 5–5.8 min (100% B), 5.8–6 min (100–1% B), and 6–6.6 min (1% B). The injection volume was 5.0 μL, and the flow rate was 0.4 mL/min. The ESI source temperature and desolvation temperature were 150 ℃ and 500 ℃ respectively. The capillary voltage was 3.0 kV in positive ion mode, and the flow rate of desolvation gas was 1000 L/h.

### 4.8. Western Blotting

VN and ileum protein lysates were subjected to immunoblotting following established protocols. Briefly, tissue-specific extracts underwent SDS-PAGE separation and semi-dry transfer to PVDF membranes. Subsequently, the membranes were incubated overnight at 4 °C with the following primary antibodies: Fos (1:1000, Cell Signaling Technology, Danvers, MA, USA), TPH (1:1000, Abcam, Cambridge, UK), 5-HT_3_A (1:1000, Abcam, Cambridge, UK), CaMKII (1:1000, Abcam, Cambridge, UK), MEK (1:5000, Cell Signaling Technology, Danvers, MA, USA), CREB (1:1000, Cell Signaling Technology, Danvers, MA, USA), ST2 (1:5000, Sanying Biology Technology, Wuhan, China), PLC-γ1 (1:1000, Abcam, Cambridge, UK), and TRPA1 (1:5000, Novus Biologicals, Centennial, CO, USA), MYD88 (1:1000, Abcam, Cambridge, UK). GAPDH (1:5000, Cell Signaling Technology, Danvers, MA, USA) was used as an internal control. All subsequent procedures were performed identically as previously detailed for the Fos protein and inflammatory factor assay. Following rapid washing by phosphate-buffered saline (PBS), cells were lysed in RIPA buffer supplemented with protease inhibitors. Total protein concentrations were determined using a BCA assay kit (CoWin Biosciences, Taizhou, China), then separated by SDS-PAGE and transferred to PVDF membranes. After blocking, the membranes were treated with primary antibodies overnight at 4 °C. After washing with TBST, they were incubated with HRP-conjugated secondary antibodies (1:5000, Cell Signaling Technology, Danvers, MA, USA) for 1 h at room temperature. The chemiluminescence detection technique was utilized to reveal protein, and ImageJ was used to quantify the protein. All Western blotting experiments were performed at least three times.

### 4.9. IFC Staining

For IFC staining, brain and ileum tissues of rats were collected. Sequential 20 μm thick coronal sections of the VN and ileum regions were prepared using a cryostat. The sections were washed with PBS three times each for 5 min and were then blocked with the PBS solution containing 0.3% Triton X-100 and 5% goat serum for 2 h at room temperature. After blocking, Sections were incubated overnight at 4 °C with the following primary antibodies: anti-Fos (1:1000, Abcam, Cambridge, UK) for VN staining and anti-TPH (1:1000, Abcam, Cambridge, UK) for ileum staining. Then, sections were washed with PBS three times each for 5 min and incubated with the corresponding secondary antibody for 2 h at room temperature. After incubation, sections were washed with PBS three times each for 5 min and DAPI was added for nuclear visualization. Images were acquired by a laser-scanning confocal microscope and analyzed using ImageJ (v2.0).

### 4.10. Immunohistochemical Staining

The sections of ileum tissue underwent deparaffinization and antigen retrieval, followed by blocking of endogenous peroxidase activity and nonspecific binding. Then, sections were incubated with a primary antibody (anti-IL-33, 1:1000, Abcam, Cambridge, UK) overnight at 4 °C, washed with PBS, and incubated with an HRP-marked secondary antibody for 50 min at room temperature. After incubation, sections were washed and added with DAB for visualization and hematoxylin for counterstaining. After mounting, a microscope and ImageJ software (v2.0) were used for observing and quantitative analysis, respectively.

### 4.11. Statistical Analysis

All data were expressed as mean ± SD and analyzed and plotted using GraphPad Prism (v9.5). One-way analysis of variance (ANOVA) was used for statistical analyses. *p*-values less than 0.05 were considered to indicate statistically significant differences.

## 5. Conclusions

In conclusion, this study elucidates the anti-MS mechanism of ZOGE through a combination of metabolomic profiling and molecular biological techniques. Our findings demonstrate that ZOGE attenuates the symptoms of MS by modulating the central vestibular MEK/CREB/Fos pathway and neurotransmission, particularly through regulation of phenylalanine, tyrosine, and tryptophan biosynthesis in the VN. Concomitantly, the mechanisms of ZOGE against MS are revealed to suppress peripheral 5-HT biosynthesis and release in the ileum via inhibition of the IL-33/ST2/PLC-γ1/TRPA1 signaling axis. These results provide evidence for the anti-MS properties of ZOGE across the gut–brain immune–neuroendocrine axis and establish a molecular basis for its potential therapeutic application in the management of MS. 

## Figures and Tables

**Table 1:** Differentially expressed metabolites in VN tissues of rats in the NC and MS groups.

No.	Metabolite	*m*/*z*	VIP	Fold Change	*p* Value	Retention Time (min)	Ion Mode
1	Xanthosine	285.0834	1.129	0.697	0.0318	3.70	ESI^+^
2	Heneicosylic acid	325.3111	1.514	0.709	0.0001	12.89	ESI^−^
3	5,8,11-Eicosatrienoic acid	305.2483	1.239	0.730	0.0041	10.82	ESI^−^
4	Erucic acid	337.3113	1.413	0.738	4.06 × 10^−6^	12.48	ESI^−^
5	N-Acetyl-D-glucosamine	222.0976	1.274	0.739	0.0008	0.89	ESI^+^
6	Sphinganine	302.3050	1.469	0.745	0.0446	9.00	ESI^+^
7	PE(O-18:0/0:0)	466.3301	1.576	0.748	0.0144	10.79	ESI^−^
8	PC(22:1/0:0)	622.4093	1.387	0.755	0.0070	11.06	ESI^−^
9	Adrenic acid	331.2640	1.124	0.755	0.0018	10.92	ESI^−^
10	LysoPC(14:0/0:0)	512.2993	1.127	0.760	0.0245	9.51	ESI^−^
11	Oleic acid	281.2482	1.146	0.770	0.0005	10.94	ESI^−^
12	Docosapentaenoic acid (22n-6)	329.2484	1.053	0.774	0.0125	10.70	ESI^−^
13	Behenic acid	339.3266	1.432	0.778	0.0003	13.46	ESI^−^
14	Homo-L-arginine	189.1348	1.050	0.780	0.0135	0.83	ESI^+^
15	Eicosadienoic acid	307.2641	1.224	0.782	0.0010	11.12	ESI^−^
16	gamma-Glutamylthreonine	249.1084	1.038	0.791	0.0016	0.89	ESI^+^
17	Tetracosanoic acid	367.3579	1.284	0.792	0.0005	14.80	ESI^−^
18	10Z-Heptadecenoic acid	267.2327	1.026	0.799	0.0143	10.64	ESI^−^
19	PC(20:4/22:6)	898.5618	1.167	0.808	0.0072	12.77	ESI^−^
20	Nervonic acid	365.3422	1.118	0.813	0.0002	13.55	ESI^−^
21	Stearic acid	283.2640	1.097	0.816	0.0002	11.50	ESI^−^
22	Arachidic acid	311.2955	1.182	0.818	3.87 × 10^−6^	12.38	ESI^−^
23	Docosatrienoic acid	333.2799	1.094	0.824	1.40 × 10^−5^	11.35	ESI^−^
24	11Z-Eicosenoic acid	309.2798	1.155	0.825	3.93 × 10^−6^	11.62	ESI^−^
25	5Z,13E-Docosadienoic acid	335.2956	1.045	0.826	3.22 × 10^−6^	11.82	ESI^−^
26	PE(16:0/0:0)	452.2781	1.033	0.826	0.0100	9.97	ESI^−^
27	PE(18:0/0:0)	480.3091	1.102	0.833	0.0453	10.45	ESI^−^
28	L-Histidine	156.0771	1.081	0.837	0.0028	0.81	ESI^+^
29	L-Arginine	175.1193	1.092	0.838	0.0026	0.80	ESI^+^
30	PE(P-18:0/0:0)	464.3142	1.283	0.844	0.0394	10.66	ESI^−^
31	L-Tyrosine	182.0815	1.076	0.854	0.0019	0.90	ESI^+^
32	L-Glutamic acid	148.0606	1.086	0.867	0.0236	0.84	ESI^+^
33	PC(16:1/22:4)	852.5771	1.083	0.907	0.0302	13.82	ESI^−^
34	Linoleyl carnitine	424.3418	1.381	1.344	0.0281	9.00	ESI^+^

## Data Availability

The original contributions presented in this study are included in the article. Further inquiries can be directed to the corresponding author.

## Supplementary Materials

The following supporting information can be downloaded at: https://www.mdpi.com/article/10.3390/ijms27146124/s1.
