# Supplementary material for: Gingerol-Rich Extract Derived from Zingiber officinale Roscoe Alleviates Motion Sickness via Inhibiting the Ileal IL-33/ST2/PLC-γ1/TRPA1 Pathway
Source: Int J Mol Sci. 2026 Jul 8;27(14):6124. doi: 10.3390/ijms27146124 (PMC13410113; doi:10.3390/ijms27146124)
Supplement: Supplementary file 1 [file ijms-27-06124-s001.zip › ijms-4342603-supplementary.pdf]

Supplementary Materials

Gingerol-Rich Extract Derived from *Zingiber officinale* Roscoe Alleviates Motion Sickness via Inhibiting the Ileal IL-33/ST2/PLC-γ1/TRPA1 Pathway

Longhui Yan †, Ziming Xia †, Yiming Luo, Junyu Bu, Kai Liang, Chang Liu, Xin Sun, Zhiyan Zhang, Min Lin, Shuchen Liu and Ying Tian *

Academy of Military Medical Sciences, Beijing 100850, China; yanlonghui111@163.com (L.Y.); zmxia22@163.com (Z.X.); 13137200855@163.com (Y.L.); bujunyu112@163.com (J.B.); a2724963261@163.com (K.L.); liuchang6926@163.com (C.L.); sunx0102@163.com (X.S.); zzyan0911@163.com (Z.Z.); limin82057@163.com (M.L.); liusc118@163.com (S.L.)

* Correspondence: tianying1977@126.com

† These authors contributed equally to this work.

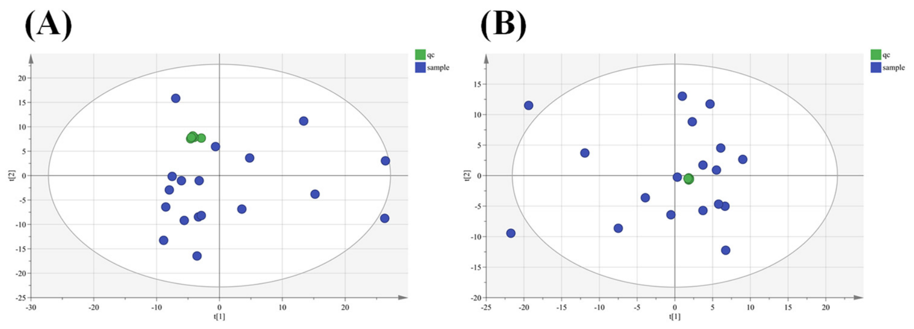

Figure S1. PCA score plots of untargeted metabolomic profiling for QC samples. Overview of PCA score plots obtained from VN samples of all experimental groups (blue) and QCs (green) in positive ion mode (**A**) and negative ion mode (**B**).

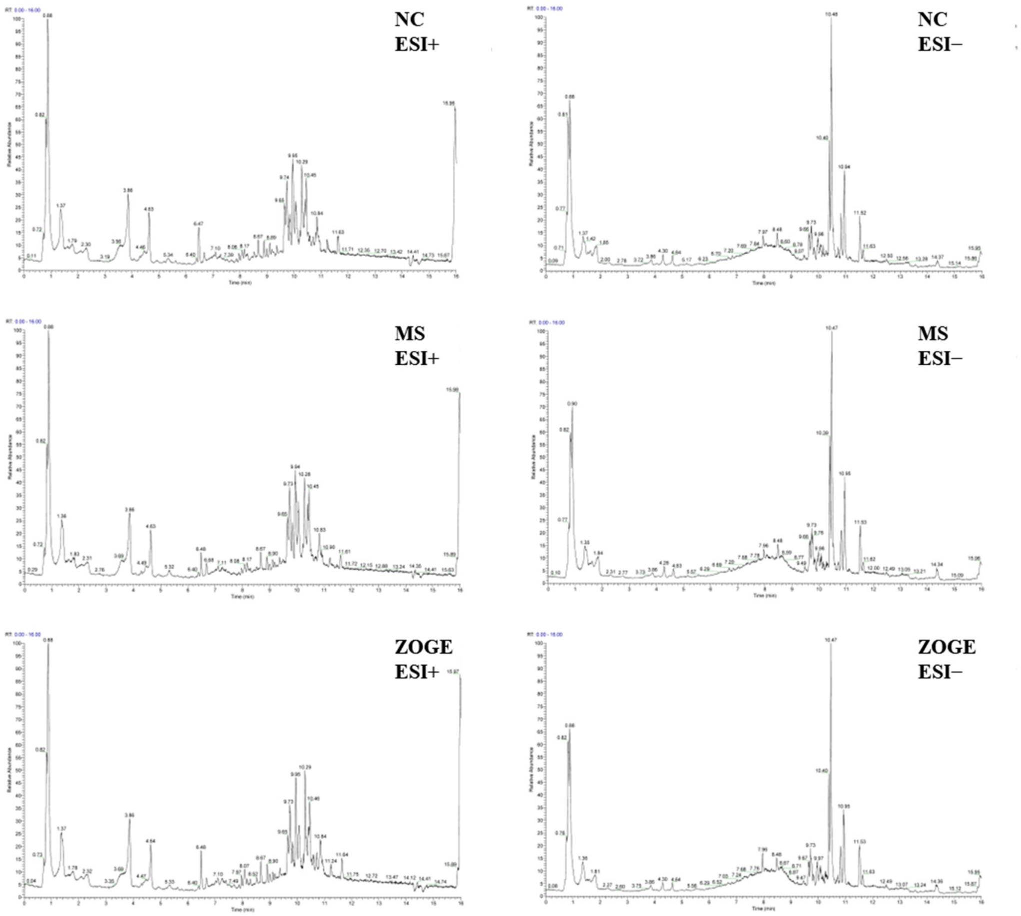

Figure S2. The TIC chromatograms based on untargeted metabolomic profiling of VN in rats.
